# A Tightly Coupled RTK/INS Algorithm with Ambiguity Resolution in the Position Domain for Ground Vehicles in Harsh Urban Environments

**DOI:** 10.3390/s18072160

**Published:** 2018-07-04

**Authors:** Wei Li, Wenyi Li, Xiaowei Cui, Sihao Zhao, Mingquan Lu

**Affiliations:** Department of Electronic Engineering, Tsinghua University, Beijing 100084, China; wli14@mails.tsinghua.edu.cn (W.L.); lia_lwy@live.com (W.L.); zsh_thu@mail.tsinghua.edu.cn (S.Z.); lumq@mail.tsinghua.edu.cn (M.L.)

**Keywords:** tightly-coupled navigation system, RTK, INS, multi-constellation, sensor integration, integer ambiguity resolution, high-precision

## Abstract

Vehicles driving in urban canyons are always confronted with a degraded Global Navigation Satellite System (GNSS) signal environment. The surrounding obstacles may cause signal reflections or blockages, which lead to large multipath noises and intermittent GNSS reception. Under these circumstances, it is not feasible to use conventional real-time kinematic (RTK) algorithms to maintain high-precision performance for positioning. In order to meet the special requirements of safety-critical applications under non-ideal observation conditions, a novel tightly coupled RTK/Inertial Navigation System (INS) algorithm is proposed in this paper, which can provide accurate and reliable positioning results continuously. Our integrated RTK/INS algorithm has three features. Firstly, INS measurements are used to help search for integer ambiguities in the position domain. INS solutions can provide a more accurate initial location and a more efficient search region. Secondly, the criterion for determining whether a candidate position is the correct solution is only related to the fractional value of the carrier-phase measurement. Thus, the new algorithm is immune to cycle slips as well as large pseudorange noises. Thirdly, our algorithm can provide more accurate ranging information than the pseudorange, even though it may not necessarily be fixed successfully, because it selects the weighted ambiguity solution as the result rather than the candidate point with maximum probability. The proposed algorithm is demonstrated on both simulated and real datasets. Compared with single epoch RTK and conventional tightly coupled RTK/INS integrations that search integer ambiguities in the ambiguity domain, our method attains better accuracy and stability in a simulated environment. Moreover, the real experimental results are presented to validate the performance of the new integrated navigation algorithm.

## 1. Introduction

With the rapid development of automatic driving as well as advanced vehicle control and safety systems in urban environments, the requirements in terms of navigation accuracy, continuity, and availability are increasing. Real-time kinematic (RTK) in surveying and mapping is a precise positioning technique by processing double-differenced carrier-phase measurements of Global Navigation Satellite System (GNSS) signals [1]. The double-difference technique can remove the error sources that are highly correlated over space and time, such as ephemeris errors. Range information with remained multipath and receiver noise can achieve cm-level accuracy only after the integer ambiguities in the carrier-phase measurements have been resolved correctly. Thus, integer ambiguity resolution (AR) becomes a critical part of the RTK technique. The conventional AR techniques are classified into three categories according to the usage of receiver measurements [2]: ambiguity resolution in the measurement domain [3], search technique in the coordinate or position domain [4], and search technique in the ambiguity domain [5,6]. The third class based on the theory of integer least-squares (ILS) is most widely used; it contains abundant techniques, such as the Fast Ambiguity Resolution Approach (FARA) [5], the Least-Squares Ambiguity Decorrelation Adjustment (LAMBDA) [6], the Least-Squares Ambiguity Search Technique (LSAST) [7]. A typical AR procedure in the ambiguity domain comprises two steps. Firstly, the float solution of ambiguities and the corresponding variance–covariance matrices are obtained by real-valued least squares estimation. Secondly, the ambiguity search process is employed to fix the integer values and adjust the baseline solution. The performance of the AR procedure in the ambiguity domain depends on a precise float estimation. Thus, it is susceptible to the number of available satellites and the quality of observations.

In harsh urban environments, the surrounding obstacles may bring two representative predicaments to the reception of satellite signals: blockages and multipath. GNSS signal blockages always make the RTK techniques suffer from a poor geometry and a reduction in the number of satellites in view. However, this dilemma has improved with the development of other constellations such as BeiDou Navigation Satellite System (BDS), which has been providing Positioning, Navigation, and Timing (PNT) services in the Asia-Pacific region since December 2012 [8]. Taking into account the use of multi-constellation systems, the number of satellites in view for users has effectively increased. Meanwhile, there is potential for the positioning accuracy and stability of RTK to be improved with the combination of GPS and BDS. In addition to solving the reduction in the total number of satellites, the ideal RTK technology also needs to deal with the frequent satellite visibility changes caused by GNSS signal blockages. For carrier-phase measurements, once the satellite signals need to be reacquired by the receiver due to changes of the availability status, the corresponding integer ambiguities have to go through a period of re-searching and fixing. In the interim, unless the fixed solution can be obtained at the time of cycle-slip occurrence, at best, only the accuracy of the float solution can be provided. For the AR techniques in the ambiguity domain, both the float solution and the success rate impose requirements on the accuracy of the pseudorange. Unfortunately, the second predicament in satellite observations under urban environments is that due to the multipath generated by the reflections on building structures, there exist large correlation errors in pseudorange measurements. Therefore, the standalone GNSS positioning methods such as RTK cannot meet the requirements under GNSS-challenged environments.

An alternative way to enhance the system performance is exploiting other sensors that are aiding. Inertial Navigation System (INS) and GNSS integrations are most widely applied because of their complementary properties. The Inertial Navigation System (INS) [9], vision system [10,11], and Wi-Fi [12] all have been take into consideration. Among the above, INS measurements are immune to external interference. They can precisely provide high-rate velocity and attitude, and give an ephemeral positioning by dead reckoning when satellite observation is absent. In turn, the accurate positioning results obtained by RTK can correct inertial errors accumulated over time. In an RTK/INS integrated navigation system, the overall accuracy still depends on the positioning result of RTK; thus, the purpose of the effective integration is to exploit inertial information to increase the search speed and success rate of the AR technique. According to the measurement model, RTK/INS integrated systems can be classified as loosely and tightly coupled structures. In the case of loosely coupled techniques, RTK and INS calculate the position and velocity results independently [13]; thus, they fail to enhance the accuracy and availability by providing additional information for the AR procedure.

By contrast, in tightly coupled RTK/INS integrations, using inertial information as an accurate position prediction in an AR procedure can effectively improve the performance of the navigation system when the satellite observation condition is poor. Conventional tightly coupled RTK/INS algorithms generally include two stages in order to ensure that their own precision is identical to the RTK results. The first stage is to use the INS measurements to assist the AR process, and the second stage is determination of whether the AR results are valid. If AR validation failed, the pseudorange measurements are utilized to correct the state variables, including position, velocity, attitude, and the biases of inertial sensors. Otherwise, the carrier-phase measurements with fixed solutions are used as more accurate ranging information to complete the correction instead.

There are two different implementations of the conventional tightly coupled RTK/INS algorithms. Both of them use an extended Kalman filter (EKF) to correct state variables and solve integer ambiguity estimation in the ambiguity domain. The first kind of tightly coupled structure is realized by fusing raw pseudorange and carrier-phase measurements as well as INS navigation solutions through an EKF; then, the float ambiguities can be estimated as filter states [14]. Subsequently, based on the information of the filter covariance matrix, a search process such as LAMBDA is performed in the ambiguity domain. The resulting fixed solution is then used to correct the remaining state variables. This implementation exploits the ambiguity constraints between adjacent epochs, making it practicable for consumer-grade receivers to achieve the desired accuracy in open sky conditions. However, adding float ambiguities to filter states also has one obvious drawback. Since the ambiguities at the previous moment affect both current state estimation and the accuracy of the carrier-phase measurement as ranging information, this algorithm is very sensitive to cycle slips in the receiver’s phase-locked loop (PLL). If the ambiguity estimation for a certain satellite is biased, then this biased estimation will affect the entire filtering process until the corresponding satellite becomes unavailable. Furthermore, in the case of frequent changes in satellite visibility, ambiguities at each GNSS epoch must be researched and fixed; thus, the benefits brought by the invariance constraint of the ambiguities will no longer exist.

In order to free the ambiguities from filter states, the second kind of tightly coupled implementation uses the Least-Squares (LS) estimation to obtain the float solution of ambiguities instead of the EKF, and its next integer search and correction steps are the same as the implementation mentioned above [15]. Compared with the AR procedure in GNSS standalone systems, not only are the pseudorange and carrier-phase measurements utilized as observables, but also the position vectors derived from the INS solution are taken as additional observables to solve this LS problem. This inertial-aided AR algorithm is expected to obtain more precise estimation and reduce the search space under INS motion constraints. Moreover, it only uses the observations of the current epoch, so the second kind of implementation is not affected by the intermittent GNSS reception. However, since both implementations search integer solutions in the ambiguity domain, the success rate of AR is unavoidably dependent on the quality of pseudorange measurements. When the pseudorange measurements have large errors due to multipath, the accuracy of the above two kinds of tight integration techniques will be degraded.

Therefore, in order to obtain high precision positioning results continuously in urban environments where satellites frequently change and large pseudorange errors exist, this paper proposes a new implementation of the tightly coupled RTK/INS integration. The new tightly-coupled algorithm proposed in this paper still contains the two stages: INS-aided AR and a correction step. However, in order to avoid the influence of large pseudorange errors in float ambiguity estimation, we perform the AR process in the Position Domain. Thus, the proposed algorithm will be abbreviated as “ARPD-RTK/INS” in the following sections. In our INS-aided AR process, the INS solution is used to provide a more accurate initial value of location and a more efficient search volume due to the use of motion constraints, when compared with the standalone-GNSS AR techniques in the position domain [4,12]. To get rid of the influence of the pseudorange error, we determine to use the posterior probability density function (PDF) to represent the weights of candidate points. Taking into account the integer property of the carrier-cycle ambiguity, the posterior PDF is only updated by the double-differenced carrier-phase measurements of GPS and BDS, which does not involve pseudorange measurements. Furthermore, we expect that a suboptimal integer solution can be obtained in the AR process, which is able to provide more precise ranging information than the pseudorange for the ensuing correction step, even if it is not necessarily the fixed integer solution. Thus, the ultimate solution is obtained by the weighted average of candidate ambiguity vectors instead of finding the fixed integer solution with the maximum probability.

This paper is organized as follows. Section 2 describes the architecture of conventional RTK/INS integrations and the transition and observation models on which to base our positioning process. Section 3 illustrates the flow chart of the new ARPD-RTK/INS algorithm, and explains the steps to solve the integer ambiguities in the position domain. Section 4 presents the experimental results of the proposed algorithm, and evaluates the performance by comparing with other algorithms on both real and simulated datasets. Section 5 summarizes our conclusions and future research directions.

## 2. The Theoretical Basis of Tightly-Coupled INS/RTK Integrations

### 2.1. Definition of State Vectors and Motion Model

In the RTK/INS integrated navigation systems, in order to clearly illustrate the positioning procedure, we first define the state vectors as well as the motion and observation models. Categorized according to different uses, there are three types of state vectors: position vector, inertial parameter vector, and ambiguity vector.

(1) $$x_{k}=(x,y,z)$$

(2) $$v_{k}{=(v}_{x}{,v}_{y}{,v}_{z},\theta ,\phi {,\psi ,b}_{a}{,b}_{g})$$

(3) $$N_{k}=(\Delta {\overset{\text{ˇ}}{N}}_{rb}^{{s,L}_{1}},\Delta {\overset{\text{ˇ}}{N}}_{rb}^{{s,L}_{2}})s=1,2,\ldots ,S_{k}$$

where *k* is the discrete-time index; $x_{k}\in {\mathbb{R}}^{3}$ is the position vector at time *k*, which only contains three-dimensional position information; and $v_{k}\in {\mathbb{R}}^{12}$ is the inertial parameter vector, which contains velocity, Euler angles, accelerometer bias, and gyroscope bias. The inertial parameter vector only exists when the navigation system integrates with the inertial navigation system. $N_{k}$ is the ambiguity vector, and $\Delta {\overset{\text{ˇ}}{N}}_{rb}^{{s,L}_{1}}$ and $\Delta {\overset{\text{ˇ}}{N}}_{rb}^{{s,L}_{2}}$ represent the single-differenced integer ambiguities of satellite *s* at *L1* and *L2* frequencies separately, obtained by the difference between rover *r* and base station *b.* $S_{k}$ is the total number of observable satellites at time *k*.

Simultaneously, the motion equation is as follows:

(4) $${(x}_{k}{,v}_{k})=f\left(x_{k-1}{,v}_{k-1}\right){+n}_{k},k=1,2,\text{…},K$$

where the function f(·) denotes the transition model, $n_{k}$ is the system noise whose distribution is independent of time. The transition model depends on the INS measurements. Using a strap-down inertial system, we need to transform the specific force vector and angular rate measured in the body frame into the Earth-centered Earth-fixed (ECEF) frame. Then, the kinematic equations are as follows [17]:

(5) $${\overset{\cdot }{C}}_{b}^{e}{=C}_{b}^{e}\Omega_{ib}^{b}-\Omega_{ie}^{e}C_{b}^{e}$$

(6) $${\overset{\cdot }{v}}_{eb}^{e}{=C}_{b}^{e}f_{ib}^{b}{+g}_{b}^{e}-{2\Omega }_{ie}^{e}v_{eb}^{e}$$

(7) $${\overset{\cdot }{r}}_{eb}^{e}{=v}_{eb}^{e}$$

where $C_{b}^{e}$ denotes the rotational transformation from the body frame to the ECEF frame, and $v_{eb}^{e}$ and $r_{eb}^{e}$ denote the Earth relative velocity and position in the ECEF frame, respectively. The equations above give their time derivatives. The vector $f_{ib}^{b}$ is the specific force that can be obtained from INS measurements directly. $g_{b}^{e}$ is the local gravity vector related to the current position $r_{eb}^{e}$. $\Omega_{ie}^{e}$ is the skew-symmetric matrix of the Earth rotation vector, and $\Omega_{ib}^{b}$ is the skew-symmetric matrix of the angular rate $\omega_{ib}^{b}$. It is noteworthy that the specific force and angular rate measurements are accompanied by noise. The simplified measurement models of the accelerometer and gyroscope are:

(8) $${\tilde{f}}_{ib}^{b}{=(I}_{3}{+M}_{a}{)f}_{ib}^{b}{+b}_{a}{+\varepsilon }_{a}$$

(9) $${\tilde{\omega }}_{ib}^{b}{=(I}_{3}{+M}_{g}{)\omega }_{ib}^{b}{+b}_{g}{+\varepsilon }_{g}$$

where ${\tilde{f}}_{ib}^{b}$ and ${\tilde{\omega }}_{ib}^{b}$ are the output vectors of inertial sensors; $\varepsilon_{a}$ and $\varepsilon_{g}$ represent the random noise; and $b_{a}$ and $b_{g}$ respectively represent the accelerometer and gyroscope biases that are contained in the state vector. The biases are modeled as constant vectors and corrected by the GNSS solutions. $I_{3}$ is the identity matrix, and $M_{a}$ and $M_{g}$ denote the matrices of the scale factor and cross-coupling errors for accelerometer and gyroscope, respectively.

### 2.2. Observation Model

Involving the new GNSS observations, this subsection discusses the definition of the observation model. At each GNSS epoch, a new observation $z_{k}$ is given by:

(10) $$z_{k}=h\left(x_{k}\right){+m}_{k},k=0,1,\text{…},K$$

The function h(·) presents the observation model. $m_{k}$ is the measurement noise whose distribution is independent of not only time but also the system noise. In the positioning process, the observation vector $z_{k}$ usually contains a dual-frequency carrier phase as well as a code pseudorange, and eliminates the noise sources that are highly correlated over space by the double-differencing method, as given by Equation (11).

(11) $$z_{k}=\left[\begin{array}{c} \nabla \Delta \Phi_{rb}^{st,L1}\\ \nabla \Delta \Phi_{rb}^{st,L2}\\ \nabla \Delta \rho_{rb}^{st,L1}\\ \nabla \Delta \rho_{rb}^{st,L2} \end{array}\right]=\left[\begin{array}{c} \frac{1}{\lambda_{1}}\left(\nabla \Delta {\hat{r}}_{rb}^{st}\right)+\nabla \Delta {\overset{\text{ˇ}}{N}}_{rb}^{st,L1}{+\varepsilon }_{\Phi ,L1}\\ \frac{1}{\lambda_{2}}\left(\nabla \Delta {\hat{r}}_{rb}^{st}\right)+\nabla \Delta {\overset{\text{ˇ}}{N}}_{rb}^{st,L2}{+\varepsilon }_{\Phi ,L2}\\ \nabla \Delta {\hat{r}}_{rb}^{st}{+\varepsilon }_{\rho ,L1}\\ \nabla \Delta {\hat{r}}_{rb}^{st}{+\varepsilon }_{\rho ,L2} \end{array}\right]$$

Here, $\nabla \Delta \Phi_{rb}^{st,L1}$ represents the double-differenced carrier-phase vector at *L1* frequency with a selected reference satellite *t*. Analogously, $\nabla \Delta \rho_{rb}^{st,L1}$ is the double-differenced pseudorange vector, and $\nabla \Delta {\overset{\text{ˇ}}{N}}_{rb}^{st,L1}$ and $\nabla \Delta {\hat{r}}_{rb}^{st}$ denote double-differenced integer ambiguities and distances, respectively. $\nabla \Delta \Phi_{rb}^{st,L2}$, $\nabla \Delta \rho_{rb}^{st,L2}$, and $\nabla \Delta {\overset{\text{ˇ}}{N}}_{rb}^{st,L2}$ are the corresponding double-differenced carrier phase, pseudorange, and integer ambiguities at *L2* frequency, respectively. ***ε*** is the measurement noise of the relevant observation.

Taking into account the clock bias between GPS and BDS, the reference satellite *t* of each constellation needs to be determined separately. Satellites at lower elevation angles are more likely to be blocked by surrounding buildings or interfered by larger atmospheric errors. To alleviate the hand-over problem, we choose the satellite with the highest elevation angle as a reference satellite.

It should be noted that the conventional tightly coupled RTK/INS algorithms often use both carrier-phase and pseudorange measurements as the observation vector when there is no fixed solution. However, the new algorithm proposed in this paper, in order to avoid the negative effects caused by large pseudorange errors, only uses the first two items of Equation (11), i.e., double-differenced carrier phases, to update state vectors.

### 2.3. Conventional Tightly Coupled RTK/INS Integrations with AR in the Ambiguity Domain

**Figure 1:**
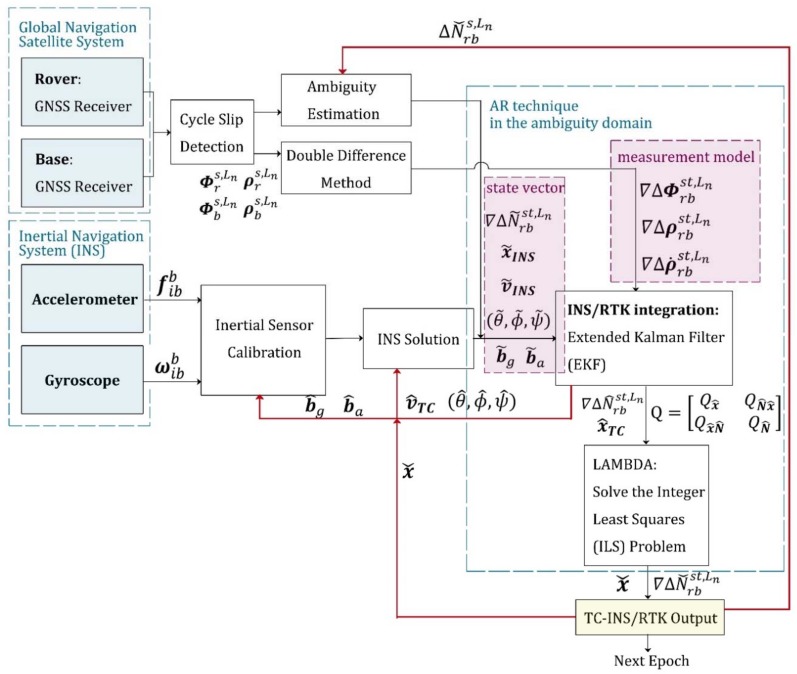
The first implementation of tightly-coupled Inertial Navigation System (INS)/real-time kinematic (RTK) integration using an extended Kalman filter (EKF).

In this subsection, we introduce two conventional RTK/INS integrated techniques, both of which complete the AR in the ambiguity domain. As we mentioned above, the typical tightly coupled RTK/INS algorithms generally include two stages: INS-aided AR, and the correction process. The total accuracy is guaranteed by the ability to successfully find a fixed solution. According to the different way that INS participates in the AR process, the existing tightly coupled algorithm is divided into two categories [10,11], as mentioned in the introduction Section 1. The first implementation is depicted in Figure 1. It uses INS measurements to help estimate float ambiguities through an EKF, and then searches for integer solutions in the ambiguity domain by LAMBDA. Compared with the ambiguity covariance matrix obtained only from the double-differenced carrier phase and pseudo-range observations, this tightly coupled algorithm takes into account a priori motion information, so the covariance matrix obtained by EKF has a more efficient search space. The filter states include position, velocity, attitude, biases of inertial sensors, and float ambiguities. Therefore, if no change in the satellite tracking status is detected, the integer solutions will be used as the initial values of filter states at the next moment, as shown by the red arrow in Figure 1. However, if there is an undetected cycle slip, the incorrect initial value can cause the subsequent filtering process to fail. At the same time, the accuracy of this tightly-coupled algorithm is affected by the quality of pseudorange measurements, which can cause a bias in the ambiguity estimation of a continuous tracking satellite.

**Figure 2:**
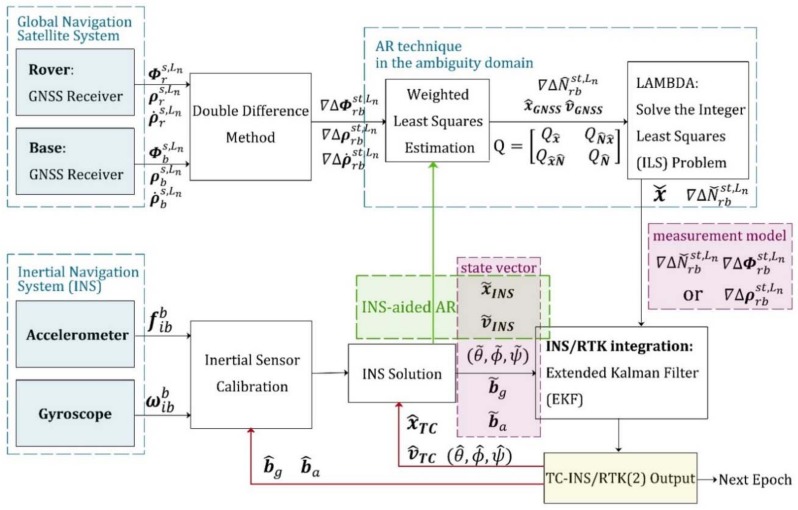
The second implementation of tightly-coupled INS/RTK integration using EKF.

The second kind of implementation is illustrated in Figure 2. Float ambiguities are obtained by least squares estimation. The next step is to search for integer solutions in the ambiguity domain and correct the state variables using the fixed carrier-phase observations. If no valid integer solution is found, pseudorange observations will be used to correct the state variables by an EKF instead. For the float ambiguity estimation process, there are not only double-differenced carrier-phase and pseudorange observations as constraints, but also the positioning result of the INS solution as additional observables, as shown by the green arrow in Figure 2. The inertial navigation assistance makes it practicable to complete the AR process when there are fewer than four satellites in a single constellation system. In contrast to the first kind of implementation, this algorithm only utilizes the current epoch observations in the AR process, so it is not affected by frequent changes in satellite visibility. From the second flowchart, it also can be clearly concluded that the quality of the pseudorange observation directly affects the success rate when searching for a fixed solution in the ambiguity domain. To avoid this undesirable influence, our new ARPD-RTK/INS algorithm searches for integer ambiguities in the position domain.

## 3. The Implementation of the Novel ARPD-RTK/INS Algorithm

### 3.1. Overall Flow Chart of the ARPD-RTK/INS Algorithm

**Figure 3:**
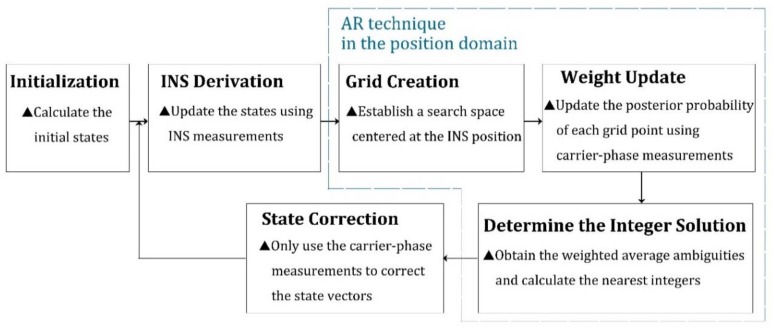
Overall flow chart of the ARPD-RTK/INS algorithm.

The flow chart of the proposed ARPD-RTK/INS algorithm that combines GPS/BDS/INS measurements is depicted in Figure 3. The new integrated navigation algorithm also has the INS-aided AR process and the correction step. To start with, the initial states are obtained by the single-epoch RTK algorithm. Then, the new states are derived from INS mechanization. This step corresponds to the “INS derivation” in Figure 3. Subsequently, critical AR steps are marked in Figure 3 with blue dashed boxes. There are two significant stages in the spatial searching procedure; the first one is to determine the searching region. We call it “grid creation”. Since the INS can provide short-term precise position prediction, the searching space is established by a set of grids centered at the estimated position. The total number of grid points is determined by the priori covariance matrix, which is obtained from the INS derivation step. The next major stage in the searching procedure is to identify which candidate grid point in the searching region is the optimal solution. The weight of each grid point depends on the distance between the float ambiguity estimation and its nearest integer vector. We complete the “weight update” step using the fractional part of carrier-phase measurements. When searching to find the optimal solution, enough candidate positions are required to avoid missing the maximum weight. As the number of candidates increases, the computational cost increases, too. Thus, our new algorithm makes a compromise between the accuracy and computational cost. The final solution is determined as the nearest integer vector of the weighted ambiguity average of ambiguities, and the state vector is corrected using the carrier ranging information corresponding to this suboptimal solution.

Since the searching step length should be proportional to the observation wavelength [16], we use wide-lane measurements to complete the AR process, although a longer wavelength is always accompanied by larger noise. If higher precision is expected, another AR process with *L1* measurements can be performed on the basis of the wide-lane positioning result. When the GNSS observation is absent, the dead reckoning approach is utilized to enhance the availability for a short time. The specific steps will be described in the following subsections.

### 3.2. State Initialization

**Figure 4:**
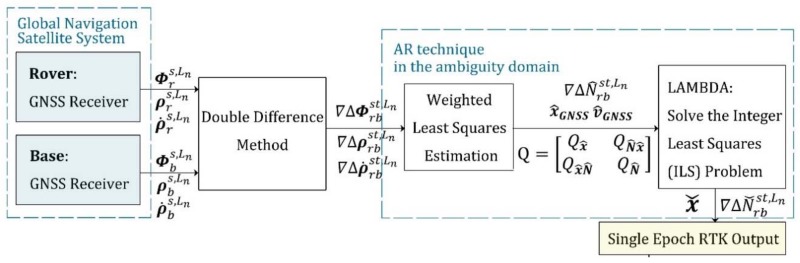
The implementation of single epoch RTK.

The first step of the ARPD-RTK/INS algorithm is to calculate the initial state vectors, including position vector $x_{0}$, inertial parameter vector $v_{0}$, and ambiguity vector $N_{0}$. We utilize the double-differenced pseudorange and carrier-phase as well as Doppler measurements to derive the initial position, velocity, and float ambiguity vectors by the weighted least squares estimation. Subsequently, the integer ambiguity solution is obtained by LAMBDA. The detailed implementation is shown in Figure 4. In the meantime, initial roll and pitch are obtained from the accelerometer measurements, while yaw is obtained from the gyroscope measurements by integrating the changes in velocity as well as orientation. The initial biases of the inertial sensors are set to zero.

### 3.3. INS Derivation

In the derivation stage, the transition model has been described in Section 2.1. Besides updating the state vectors, their corresponding error propagation also needs to be calculated. The state error vectors of the ARPD-RTK/INS algorithm contain the following 15 states.

(12) $$\delta E=\left[\begin{array}{c} {\delta \psi }_{eb}^{e}\\ {\delta v}_{eb}^{e}\\ {\delta r}_{eb}^{e}\\ b_{a}\\ b_{g} \end{array}\right]$$

where ${\delta \psi }_{eb}^{e}$ denotes the error of the Euler angle from the ECEF frame to the body frame, and ${\delta v}_{eb}^{e}$ and ${\delta r}_{eb}^{e}$ denote the errors of velocity and position, respectively. They describe a kinematic property of the body frame with respect to the ECEF frame, which is expressed in ECEF frame axes. Then, the error propagation in the ECEF frame over the time interval $\tau_{s}$ can be obtained by the time derivative of each state error as follows [17]:

(13) $$\Phi_{INS}^{e}\approx \left[\begin{array}{ccccc} I_{3\times 3}{-\Omega }_{ie}^{e}\tau_{s} & 0_{3\times 3} & 0_{3\times 3} & 0_{3\times 3} & C_{b}^{e}\tau_{s}\\ F_{21}^{e}\tau_{s} & I_{3\times 3}{-2\Omega }_{ie}^{e}\tau_{s} & F_{23}^{e}\tau_{s} & C_{b}^{e}\tau_{s} & 0_{3\times 3}\\ 0_{3\times 3} & I_{3\times 3}\tau_{s} & I_{3\times 3} & 0_{3\times 3} & 0_{3\times 3}\\ 0_{3\times 3} & 0_{3\times 3} & 0_{3\times 3} & I_{3\times 3} & 0_{3\times 3}\\ 0_{3\times 3} & 0_{3\times 3} & 0_{3\times 3} & 0_{3\times 3} & I_{3\times 3} \end{array}\right]$$

(14) $$F_{21}^{e}=[-\left(C_{b}^{e}f_{ib}^{b}\right)\wedge ]$$

(15) $$F_{23}^{e}=-\frac{{2\gamma }_{ib}^{e}}{r_{\mathrm{eS}}^{e}}\cdot \frac{r_{eb}^{e}{}^{T}}{\left|r_{eb}^{e}\right|}$$

The function [x∧] represents the skew-symmetric matrix of x. $\gamma_{ib}^{e}$ denotes the acceleration due to the gravitational force, and $r_{\mathrm{eS}}^{e}$ is the geocentric radius at the Earth’s surface. The remaining notations are identical with the ones mentioned above.

### 3.4. Grid Creation

After obtaining the INS derived position and the error covariance matrix, we must establish the spatial search region centered on the current position. A simplified approach is to draw a set of fixed-distance grid points, and each point corresponds to a set of integer ambiguities. Considering the integer property of ambiguities, the weight of a grid point is negatively correlated with the residual between its float ambiguity solution and the nearest integer vector. For wide-lane observations, the appropriate distance between the two nearest grid points is determined by the satellite elevation distribution, which has been discussed in detail in earlier work [18]. Thus, we still choose 0.2 m as the constant distance.

**Figure 5:**
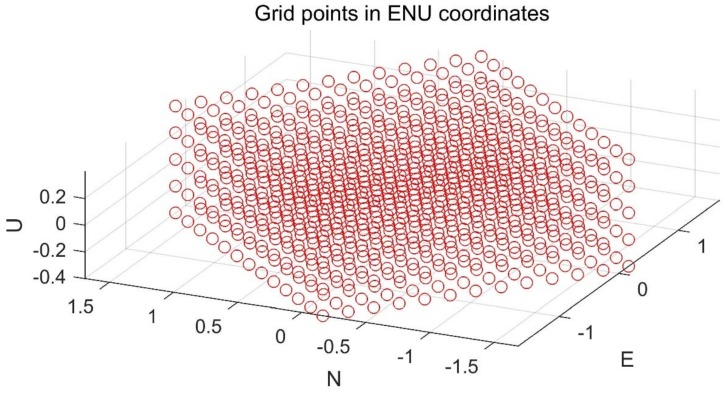
A typical example for the grid creation step.

Since the error distribution of the vehicle position in the ECEF frame is obtained by the previous step, and the ground vehicle moves relatively slowly in the height direction, using such a priori information can make the grid point allocation more efficient. The error distribution needs to be converted from the ECEF frame to the ENU frame. In order to avoid the influence of position error covariance, we refer to the idea of “decorrelation” in the ambiguity domain when determining the distribution directions of the grid points. In the horizontal plane, grid points are created along the major and minor axes of the error ellipse. A typical example for the grid creation is depicted in Figure 5.

### 3.5. Weight Update

The purpose of the weight update step is to identify the correct candidate position. Taking the ambiguity function method (AFM) as an example [4], it introduced an ambiguity function and aimed at finding the maximum value. The adaptive ambiguity function, which uses trigonometric functions to process the fractional value of carrier-phase measurement, is insensitive to the whole-cycle change of the carrier-phase measurement. We expect to maintain the advantage of this objective function, and determine to use the posterior probability density function (PDF) to replace the multi-peak and complicated ambiguity function as a new objective function. From the Bayesian rule, the posterior density can be factorized by:

(16) $$P\left(x_{k}{|z}_{k}\right)=\frac{P\left(z_{k}{|x}_{k}\right){P(x}_{k})}{\int P\left(z_{k}{|x}_{k}\right){dx}_{k}}$$

The prior distribution ${P(x}_{k})$ can be obtained from the INS motion model. Assuming that the system noise conforms to the Gaussian distribution, the prior distribution ${P(x}_{k})$ can be achieved as:

(17) $${P(x}_{k})\propto exp\left[-\frac{1}{2}{\left(x_{k}-{\hat{x}}_{INS}\right)}^{T}{C(x}_{k}{)}^{-1}\left(x_{k}-{\hat{x}}_{INS}\right)\right]$$

where ${\hat{x}}_{INS}$ denotes the position derived by INS measurements, and ${C(x}_{k})$ is the position error covariance matrix.

When new GNSS measurements arrive, the calculation of likelihood distribution $P\left(z_{k}{|x}_{k}\right)$ is similar to Equation (17) under the assumption that the measurement noise is Gaussian. Since we try to avoid involving pseudorange observations, the update of the grid weights only makes use of current carrier phase measurements. For a certain grid point, the float ambiguity corresponding to each satellite can be calculated according to the position, and there definitely exists a set of nearest integer solutions. The float double-difference ambiguity can be estimated by:

(18) $$\nabla \Delta N_{rb}^{st,WL}=\nabla \Delta \Phi_{rb}^{st,WL}-\frac{1}{\lambda_{WL}}[\left({\hat{r}}_{\mathrm{rs}}-{\hat{r}}_{\mathrm{bs}}\right)-\left({\hat{r}}_{\mathrm{rt}}-{\hat{r}}_{\mathrm{bt}}\right)]$$

where $\lambda_{WL}$ is the wide-lane wavelength, ${\hat{r}}_{\mathrm{rs}}$ is the estimated distance from rover *r* to satellite *s*, and ${\hat{r}}_{\mathrm{bs}}$ is the estimated distance from base station *b* to satellite *s*. The integer ambiguity solution is set as:

(19) $$\nabla \Delta {\overset{\text{ˇ}}{N}}_{rb}^{st,WL}=R(\nabla \Delta N_{rb}^{st,WL})$$

where the function R(·) denotes its nearest integer. Consequently, the likelihood distribution $P\left(z_{k}{|x}_{k}\right)$ is proportional to:

(20) $$P\left(z_{k}{|x}_{k}\right)\propto exp\left[-\frac{1}{2}{\left(\nabla \Delta N_{rb}^{st,WL}-\nabla \Delta {\overset{\text{ˇ}}{N}}_{rb}^{st,WL}\right)}^{T}{C(N}_{WL}{)}^{-1}\left(\nabla \Delta N_{rb}^{st,WL}-\nabla \Delta {\overset{\text{ˇ}}{N}}_{rb}^{st,WL}\right)\right]$$

The error covariance matrix ${C(N}_{WL})$ can be simplified as a constant matrix, which is composed of the covariance matrix of measurement noise $R_{MEAS,WL}$ and single-differencing matrix ***D***.

(21) $${C(N}_{WL})=\left[\begin{array}{c} {\mathrm{DR}}_{\Phi ,WL}D^{T} \end{array}\right]$$

(22) $$D=\left[\begin{array}{ccccc} -1 & 0 & \cdots & 0 & 1\\ 0 & -1 & \cdots & 0 & 1\\ \vdots & \vdots & \ddots & \vdots & \vdots \\ 0 & 0 & \cdots & -1 & 1 \end{array}\right]$$

The required posterior probability:

(23) $$P\left(x_{k}{|z}_{k}\right)\propto {P(x}_{k})\cdot P\left(z_{k}{|x}_{k}\right)$$

Each grid point can calculate its posterior probability according to Equation (23). We use this value as the weight of the corresponding candidate position. However, the grid point with the largest weight value is not supposed to be the integer ambiguity solution. Instead, we use the weighted integer ambiguity vector to provide ranging information, which is more accurate than the pseudorange, but is not necessarily a fixed solution that can be verified by an ambiguity evaluation such as a ratio test.

### 3.6. Determine the Integer Solution and the Correction Step

As we discussed above, the ultimate ambiguity solution can be calculated by:

(24) $$\nabla \Delta {\overset{\text{ˇ}}{N}}_{rb}^{st,RES}=R\left(\sum P\left(x_{k}{|z}_{k}\right)\cdot \nabla \Delta {\overset{\text{ˇ}}{N}}_{rb}^{st,WL}\right)$$

**Figure 6:**
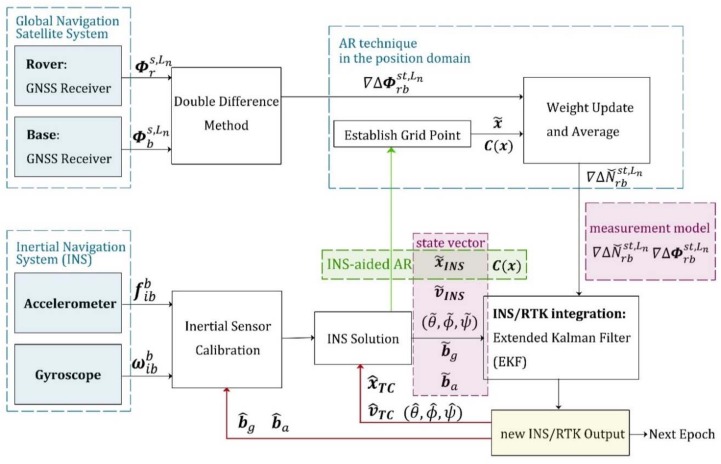
The implementation of the ARPD-RTK/INS algorithm.

Using carrier-phase measurements with suboptimal integer ambiguities may attain more accurate positioning results than the float AR solutions or pseudorange measurements. The following correction step is identical to the second types of tightly coupled RTK/INS integration described in Section 2.3. The implementation of the ARPD-RTK/INS algorithm is depicted in Figure 6.

## 4. Experimental Results and Discussion

To evaluate the performance of the proposed ARPD-RTK/INS algorithm, experiments are conducted on both simulated and real datasets. The experiment mainly compares the accuracy of the following algorithms under different conditions:

1. Single-epoch RTK: Only the current satellite observations are utilized to perform an integer ambiguity resolution at each epoch. The AR technique that we use here is the LAMBDA method. The detailed implementation is depicted in Figure 4.
2. The first kind of tightly coupled RTK/INS integration: Raw carrier-phase measurements, pseudorange measurements, and inertial information are fused to estimate the float ambiguities using EKF. Then, the integer solution is searched for in the ambiguity domain. The Kalman filter states contain position, velocity, attitude, the biases of inertial sensors, and float ambiguities. The detailed implementation is depicted in Figure 1. In this section, this algorithm is abbreviated as “TC-RTK/INS (1)”.
3. The second kind of tightly coupled RTK/INS integration: Least-Squares (LS) estimation is used to obtain the float ambiguities. In order to solve this LS problem, not only pseudorange and carrier-phase measurements, but also the position provided by the INS are utilized as constraints. The detailed implementation is depicted in Figure 2. In this section, this algorithm is abbreviated as “TC-RTK/INS (2)”.
4. The proposed ARPD-RTK/INS algorithm: Different from the above three positioning methods, the new algorithm obtains the integer ambiguity solution by searching in the position domain, which is not affected by the quality of pseudorange measurements. The detailed implementation is depicted in Figure 6.

### 4.1. Experiments on Simulation Datasets

We use Matlab to simulate the motion process of a ground vehicle, including varying accelerated motion and yaw rotations. The average speed during the 30-min simulation period is 3.67 m/s. Based on the true trajectory, the INS measurements were generated at 100 Hz. The measurement models of accelerometer and gyroscope are described in Equations (8) and (9). Here, we use the NovAtel SPAN-CPT system as a reference for the determination of inertial navigation parameters. Its specifications are given in Table 1 [15].

In our simulation, the constant bias of the accelerometer was set as a three-component vector in the body frame as follows:

(25) $$b_{a}=\left(45,-33,40\right)mg$$

The constant bias of the gyroscope in the body frame was:

(26) $$b_{g}=\left(20,-20,20\right){}^{\circ}/\mathrm{hr}$$

The random noise was drawn from a zero-mean Gaussian distribution, the root power spectral density (PSD) of the accelerometer and gyroscope were set as follows, respectively:

(27) $$\sigma_{a}=0.55\mathrm{mg}/\sqrt{\mathrm{Hz}}$$

(28) $$\sigma_{g}=0.00667{}^{\circ}/\sqrt{\mathrm{hr}}$$

The scale factor and cross-coupling errors of the accelerometer and gyroscope were:

(29) $$M_{a}=\left[\begin{array}{ccc} 2500 & -750 & 500\\ -375 & -3000 & 625\\ -625 & 250 & 1000 \end{array}\right]\mathrm{ppm}$$

(30) $$M_{g}=\left[\begin{array}{ccc} 1000 & -400 & 300\\ 0 & -800 & -210\\ 0 & 0 & -430 \end{array}\right]\mathrm{ppm}$$

Besides supplying consumer-grade inertial sensor measurements, GNSS measurements were generated at 1 Hz. Our simulation of satellite motion as well as GNSS pseudorange and carrier phase observations were also implemented in Matlab. To obtain ECEF Cartesian positions and the velocities of simulation satellites in the constellation, we use the matlab program created by Paul Groves [17] for reference. It assumes that the satellites are evenly distributed on a circular orbit. When generating GNSS pseudorange observations, we considered ionosphere errors, troposphere errors, ephemeris errors, satellite clock errors, receiver noise, and multipath. When generating the GNSS carrier phase, the receiver noise and multipath are set to smaller values. In order to test the performance of the proposed ARPD-RTK/INS algorithm under different conditions, we designed an experimental group and a control group with different satellite visibility, as listed hereafter:

- Scene A: Control group. From 0 s to 1800 s, there are sufficient satellite measurements with no cycle slips occurring.
- Scene B: Experimental group. From 0 s to 1800 s, the total number of satellites in view is more than four, but there exist frequent changes in satellite visibility.

**Figure 7:**
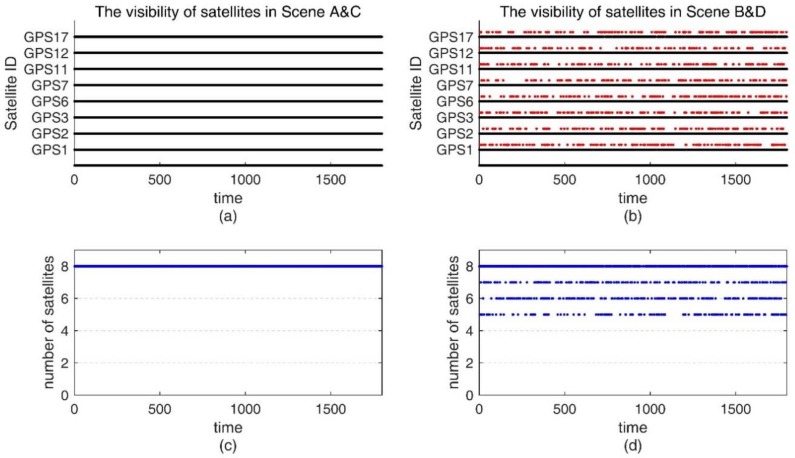
(**a**) The satellite visibility of the 1800-s simulation data for the control group; (**b**) The satellite visibility for experimental group; (**c**) The number of satellites in Scene A and C; (**d**) The number of satellite in Scene B and D.

The visibility of satellites is shown in Figure 7. In Figure 7a,b, the red points represent invisible satellites, and the black points represent available observations. Figure 7c,d depict the number of visible satellites for the control and experimental groups, respectively.

**Figure 8:**
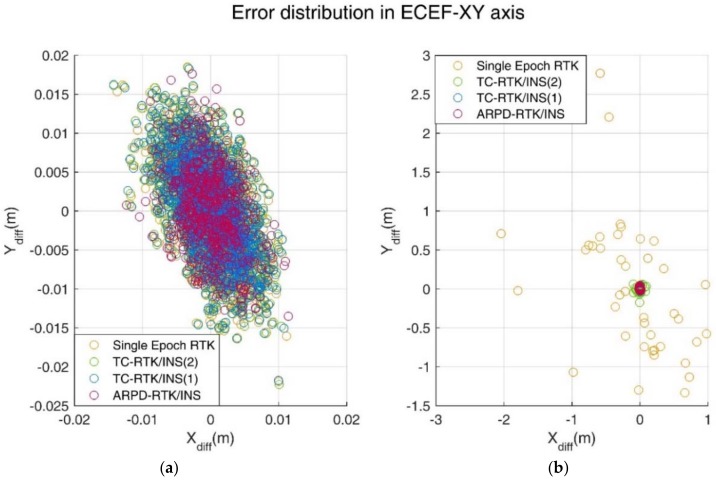
(**a**) Error distribution in Earth-Centered Earth-Fixed (ECEF)-XY axis under Scene A; (**b**) Error distribution in ECEF-XY axis under Scene B.

In the simulation environment, the standard deviation of the carrier phase noise is 5 mm, and the standard deviation of the pseudorange noise is 30 cm. Figure 8 shows the position errors of different RTK solutions in the ECEF frame. Table 2 compares the accuracy and fix rate of these algorithms numerically.

By analyzing the error results in Table 2, we can conclude as follows.

- By comparing the positioning results of the four methods under Scene A, we can find that a similar performance can be obtained by four different methods when under good satellite observation conditions. The maximum position error is about 3 cm, which meets the desired high-precision requirement.
- The number of available satellites plays an important role in the accuracy of the positioning algorithm. Comparing the single epoch RTK results in Scene A and Scene B, it is found that the fix rate decreases as the number of satellites decreases. Even if additional inertial information is considered in the AR process, such as the TC-RTK/INS (2) algorithm, the final fix rate is also affected by the number of visible satellites. For the other two algorithms that do not involve the fix rate change, the overall accuracy also slightly degraded due to a reduction in the number of satellites.
- Comparing the performance of the four algorithms in Scene B, methods 1 and 3 both show that the fix solution cannot be obtained all of the time. It can be seen that the AR process with INS-aiding has a higher fix rate than the single-epoch RTK. When the AR process failed, the positioning accuracy will be degraded, which depends on the quality of the pseudorange observation. Since we assume that all of the cycle slips can be detected before the positioning algorithm is performed, even in the scene where the satellite observability frequently changes, the TC-RTK/INS (1) algorithm will not be influenced by the wrong ambiguity and remain the ideal positioning accuracy. The reason why the ARPD-RTK/INS and TC-RTK/INS (1) algorithm perform better than the other two algorithms is that the position predicted by the INS measurement is more accurate than the position derived from the pseudorange estimation, so that a more effective ambiguity search region can be obtained.

**Figure 9:**
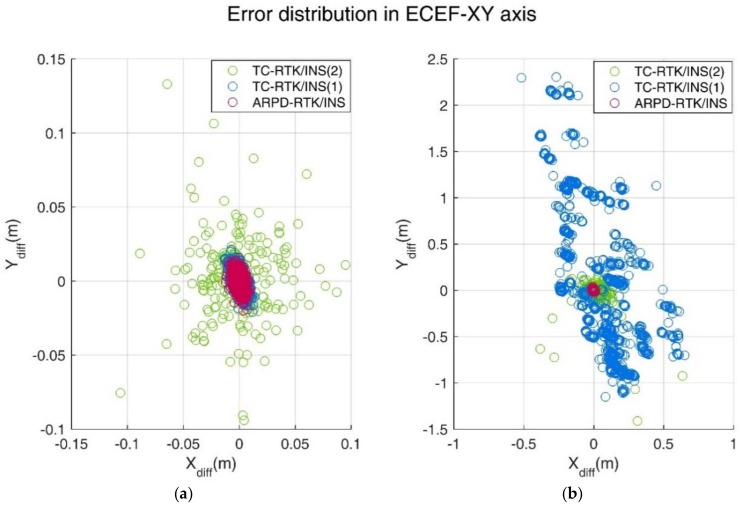
(**a**) Error distribution in ECEF-XY axis under Scene C; (**b**) Error distribution in ECEF-XY axis under Scene D.

If we want to embody the advantages of the ARPD-RTK/INS over the TC-RTK/INS (1), one way is to add cycle slips that cannot be detected before performing the positioning algorithm. As a result, the TC-RTK/INS (1) algorithm, which contains float ambiguities in the filter states, will deviate from the true value during the filtering process. Another way is to change the quality of pseudorange observations. Thus, more observation noise is added to the pseudorange measurements while keeping the satellite visibility of Scene A and Scene B unchanged. The new scene is defined as Scene C and Scene D, where the standard deviation of carrier-phase noise is still 5 mm, and the standard deviation of pseudorange noise becomes 3 m. The differences between the corresponding positioning results and the true values are shown in Figure 9 and Table 3.

In theory, except for the ARPD-RTK/INS algorithm, the positioning results of the remaining algorithms are all related to the quality of the pseudorange observations. When the pseudorange noise is large, it can cause errors when searching in the ambiguity domain. If satellites can be observed continuously, TC-RTK/INS (1) can maintain good accuracy due to the constraints of the correct fixed solution at the initial time. However, the algorithms that need to search for fixed solutions at each epoch, such as single-epoch RTK and TC-RTK/INS (2), will show a significant drop in the fix rate. The advantages of the new ARPD-RTK/INS algorithm are reflected when some of the satellites need to be re-tracked. At this time, the TC-RTK/INS (1) algorithm will suffer from the biased estimation of new ambiguities due to the influence of pseudorange errors, and this estimation will be reserved during the subsequent filtering process.

**Figure 10:**
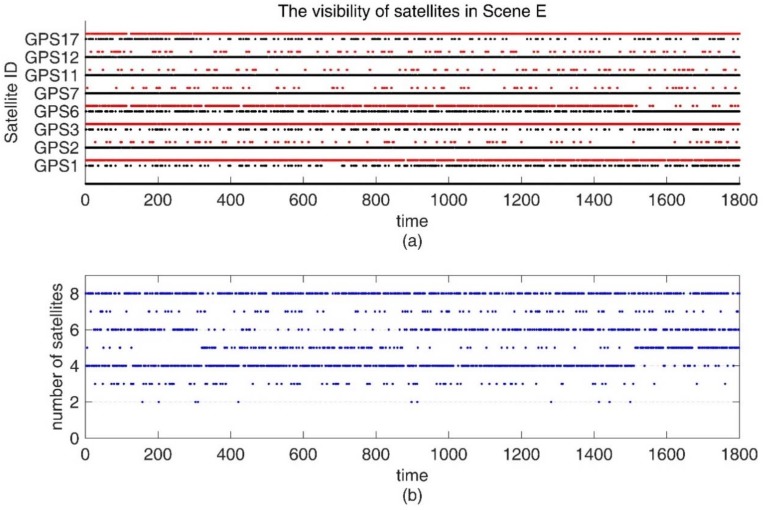
(**a**) The satellite visibility of the 1800 s simulation data for Scene E; (**b**) The number of satellites in Scene E.

**Figure 11:**
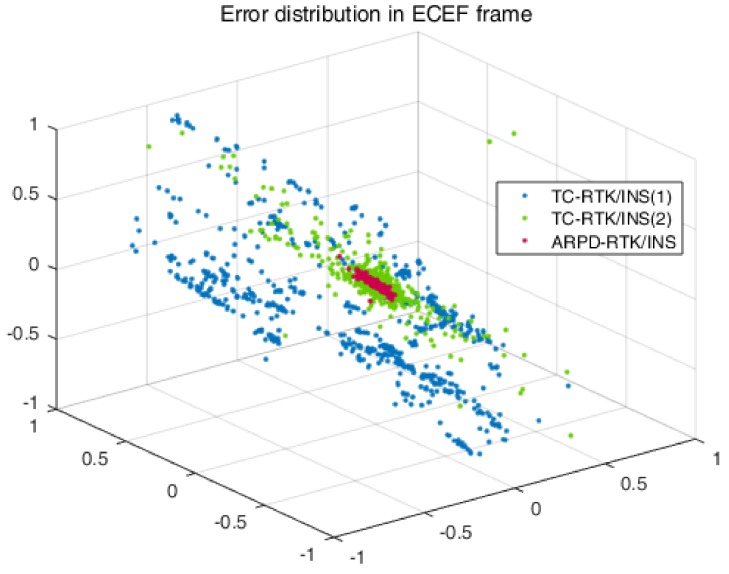
Error distribution in ECEF-XYZ axis.

In addition, we also conducted experiments in an environment with insufficient satellites. From 0 s to 1800 s, there exist frequent satellite changes, and there is no guarantee that there are more than five visible satellites. The standard deviation of the carrier phase noise is 5 mm, and the standard deviation of the pseudorange noise is 30 cm. The visibility of the satellites is shown in Figure 10. In this condition, only the tightly coupled RTK/INS integrations can obtain the positioning results continuously. From the position errors shown in Table 4 and Figure 11, we can conclude that as long as the number of visible satellites is not less than three, the ARPD-RTK/INS algorithm can obtain the available integer ambiguity solutions near the position predicted by INS. When the number of satellites is only two or less, it can only rely on the short-term positioning results of dead reckoning. Therefore, the overall accuracy is lower than the above four scenes, but it can still maintain decimeter-level accuracy.

**Figure 12:**
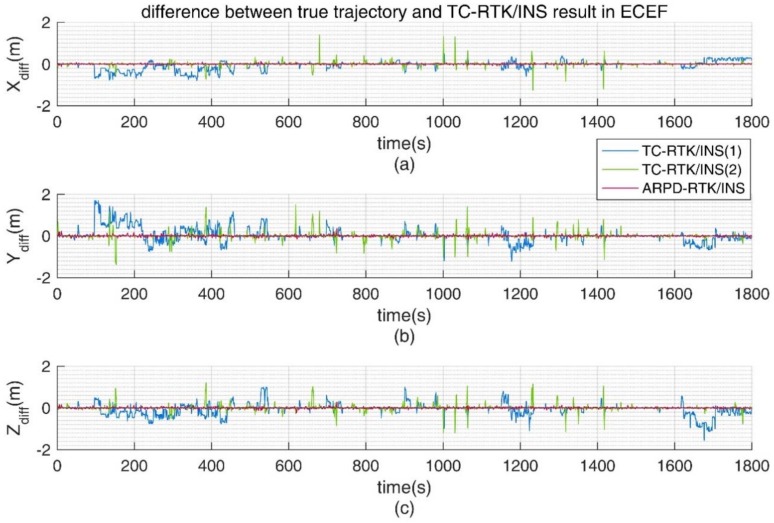
(**a**) Error distribution in Earth-Centered Earth-Fixed (ECEF)-XYZ axis for different algorithms; (**b**) Error distribution in ECEF-Y axis; (**c**) Error distribution in ECEF-Z axis.

For TC-RTK/INS (1), when the number of visible satellites is small, a biased ambiguity estimation may be obtained. If the corresponding satellite is still being tracked continuously, the biased ambiguity will remain in the filter and cause the positioning result to shift, as shown by the blue line in Figure 12. For TC-RTK/INS (2), although the float ambiguity solution can be obtained with the aid of INS when the number of satellites is small, the fix rate is lower than 70%. Thus, this algorithm can only maintain the precision of the pseudorange most of the time.

### 4.2. Real Experimentation

**Figure 13:**
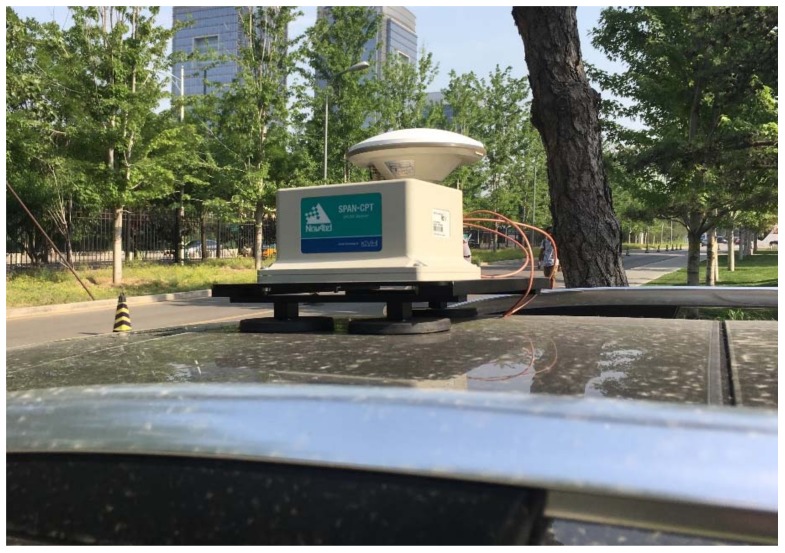
The experimental equipment for data collection.

The real experimental results are presented to validate the performance of the proposed algorithm. A ProPak6 GNSS Receiver was used as a base station and placed on the rooftop of the Weiqing Building, Tsinghua University, Beijing, China. It provided double-frequency pseudorange, Doppler, and carrier-phase measurements at 1 Hz. The dataset of the rover station was collected by a moving vehicle equipped with NovAtel SPAN-CPT, which was composed of a GNSS receiver and an Inertial Measurement Unit (IMU). The experimental equipment for data collection is shown in Figure 13. GNSS measurements were also output at 1 Hz, and inertial information including the specific force and angular rate measurements were collected at 100 Hz. The vehicle was driven around the Tsinghua campus with a maximum speed of 57.18 km/h. The baseline was less than 2 km.

**Figure 14:**
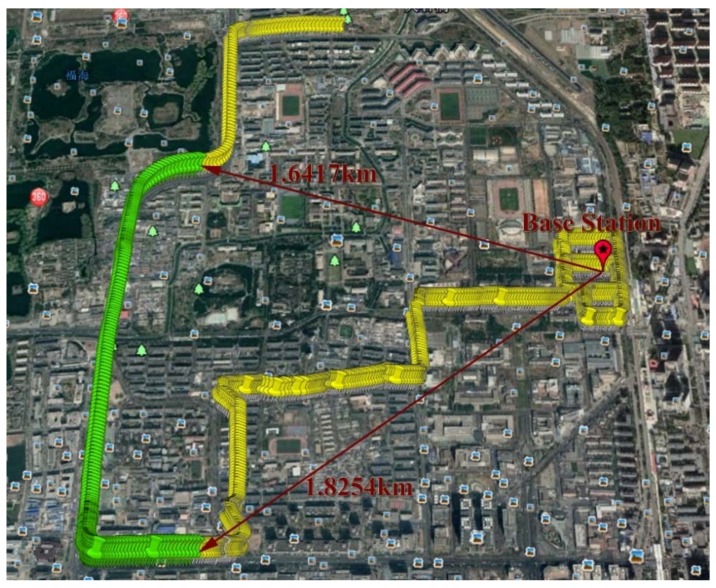
The entire path obtained by Inertial Explorer as a reference (from Google Earth).

**Figure 15:**
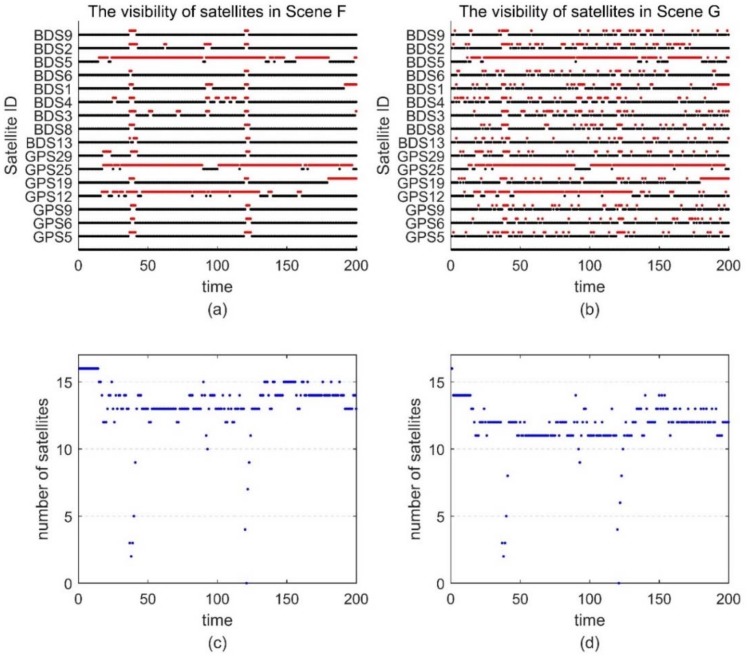
(**a**) The satellite visibility of the 200-s real data for Scene F; (**b**) The satellite visibility for Scene G; (**c**) The number of satellites in Scene F; (**d**) The number of satellites in Scene G.

Since the ground truth is unknown, we select a segment with better satellite conditions and use the result of the post-processing software Inertial Explorer [19] as the reference trajectory to compare with the ARPD-RTK/INS algorithm. The reference trajectory is marked by a set of yellow points in Figure 14, and the selected segment is marked by green points. During the 200-s data, the satellite visibility is indicated in the left part of Figure 15. The red points represent invisible satellites, while the black points represent the available satellites in Figure 15a. Therefore, we can see that the satellite observations are in good condition, except for the periods around the 40th and 121st s.

In order to compare the performance of the different positioning algorithms, we add cycle slips and large pseudorange noises under the existing observations artificially. Different scenes are designed as follows:

- Scene F: The original real dataset.
- Scene G: Frequent satellite changes are added to the original dataset.
- Scene H: Large pseudorange noise are added to the original dataset. The standard deviation of the additional noise is 3 m. Meanwhile, the number of visible satellites is reduced, and only the BDS observations are preserved.
- Scene I: Frequent satellite changes are added to the Scene H. At the same time, low-elevation satellites are removed.

**Figure 16:**
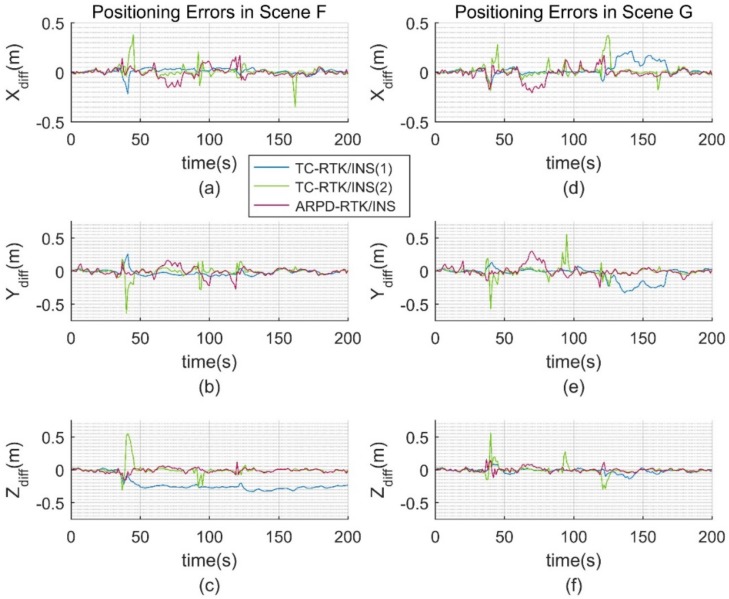
(**a**) Error distribution in the ECEF-X axis for Scene F; (**b**) Error distribution in the ECEF-Y axis for Scene F; (**c**) Error distribution in the ECEF-Z axis for Scene F; (**d**) Error distribution in the ECEF-X axis for Scene G; (**e**) Error distribution in the ECEF-Y axis for Scene G; (**f**) Error distribution in the ECEF-Z axis for Scene G.

**Figure 17:**
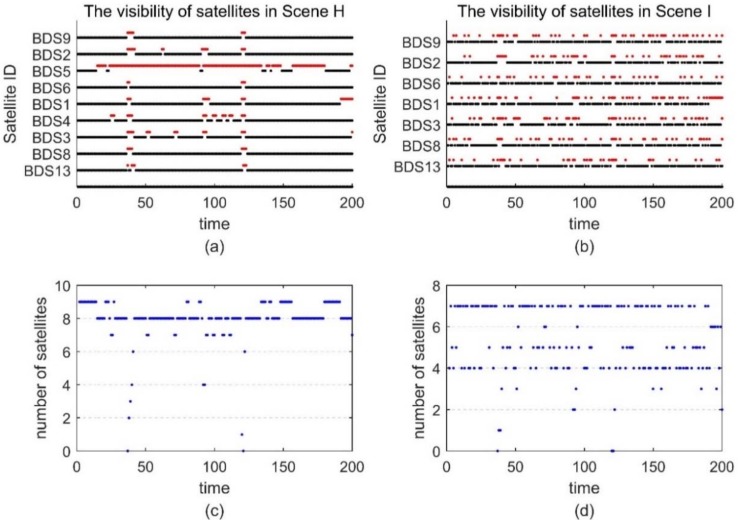
(**a**) The satellite visibility of the 200-s real data for Scene H; (**b**) The satellite visibility for Scene I; (**c**) The number of satellites in Scene H; (**d**) The number of satellites in Scene I.

**Figure 18:**
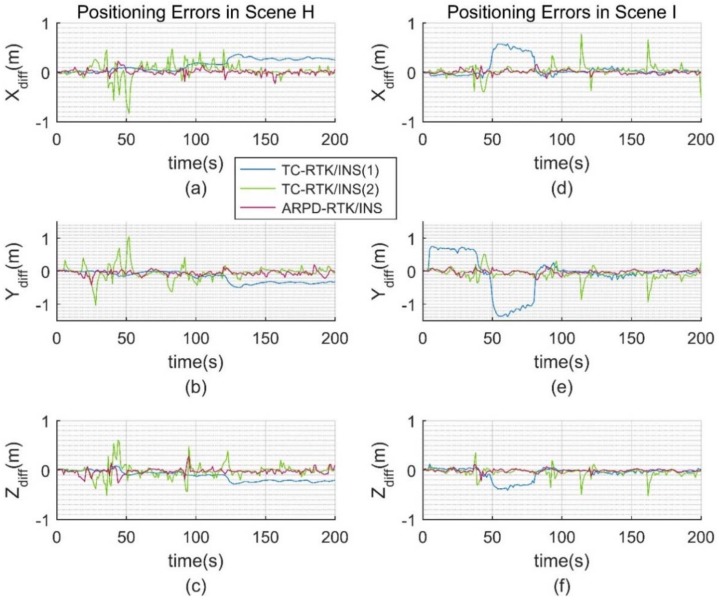
(**a**) Error distribution in the ECEF-X axis for Scene H; (**b**) Error distribution in the ECEF-Y axis for Scene H; (**c**) Error distribution in the ECEF-Z axis for Scene H; (**d**) Error distribution in the ECEF-X axis for Scene I; (**e**) Error distribution in the ECEF-Y axis for Scene I; (**f**) Error distribution in the ECEF-Z axis for Scene I.

The satellite visibility of scene F and G is shown in Figure 15. The position errors of different tightly coupled RTK/INS algorithms in the ECEF frame are shown in Figure 16. Similarly, the satellite visibility of scene H and I is shown in Figure 17, and its corresponding position errors are shown in Figure 18. Table 5 and Table 6 compare the accuracy and fix rate of these algorithms numerically.

The conclusions obtained from the real datasets are basically consistent with those from the simulation datasets. In the original scene F, when the integer ambiguity solution of all of the satellites needs to be resolved again, the advantage of the TC-RTK/INS (1) method putting the floating ambiguity into the filter state will no longer exist. As shown in Figure 16c, if the ambiguity estimation is biased, this error will continue until the corresponding satellite becomes invisible. As for the TC-RTK/INS (2) method, it is clear that the algorithm has poor performance when the number of available satellites is small. The reason is that obtaining a fixed solution in this case is highly dependent on the satellite geometry and the quality of pseudorange observations. If the AR process fails, the TC-RTK/INS (2) algorithm can only degenerate to the accuracy of pseudorange. Our ARPD-RTK/INS algorithm uses a weighted average instead of a fixed solution, so it can always provide a more precise ranging measurement than the pseudorange. This advantage is reflected in Table 5 and Table 6.

When the pseudorange noise is added to the real dataset, the ARPD-RTK/INS algorithm can still maintain submeter-level accuracy. The maximum difference between the position results of the proposed algorithm and Inertial Explorer is less than 0.5 m in GNSS-challenged environments. This demonstrates the practicability of the new algorithm in the real environment. However, the fix rate of the other two tightly coupled algorithms decreases with the increasing pseudorange noise. The blue lines in Figure 18a–c show the negative effect of biased ambiguity estimation in the filtering process. It can be concluded that the ARPD-RTK/INS algorithm performs better than the traditional RTK/INS integrated algorithms in terms of accuracy and stability, especially in environments with large pseudorange errors.

## 5. Conclusions

This paper proposes the ARPD-RTK/INS algorithm, which searches the integer ambiguities in the position domain and correct positioning states by carrier-phase measurements. The purpose is to provide a continuous and accurate positioning result under harsh GNSS signal conditions. Compared with the conventional tightly coupled RTK/INS integrations, the new algorithm has three distinct advantages. Since we search the integer ambiguity solution in the position domain, the ARPD-RTK/INS is insensitive to frequent cycle slips. The weight of the candidate position is only related to the fractional part of the carrier-phase measurements, so the second advantage is that our algorithm is not interfered by pseudorange errors. Last but not least, the new method can provide relatively accurate ranging information steadily, because it uses weighted average ambiguity results to avoid the risk of failure.

Our future work aims to improve the efficiency of the ARPD-RTK/INS algorithm. One direction is to use pre-integration during the INS process. Another direction is to use the sequential importance sampling (SIS) technique instead of uniform sampling. If the amount of computation is effectively reduced, the ARPD-RTK/INS algorithm can be applied more widely in the future.

## Figures and Tables

**Table 1:** Performance specifications of SPAN-CPT.

Fiber Optic Gyros		Micro-Electro-Mechanical System (MEMS) Accelerometers	
Bias	20°/hr	Bias	50mg
Angular Random Walk	$0.00667{}^{\circ}/\sqrt{\mathrm{hr}}$	Bias Stability	±0.75mg
Scale Factor	1500 ppm	Scale Factor	4000 ppm

**Table 2:** Trajectory error and fix rate for different RTK algorithms with small pseudorange errors (Unit: cm). RMSE: root mean square error.

Algorithm	Conditional Control Group A			Experimental Group B		
	Max Error	RMSE	Fix Rate	Max Error	RMSE	Fix Rate
Single Epoch RTK	2.62	0.85	99.83%	321.77	20.66	92.78%
TC-RTK/INS (1)	2.59	0.85	100%	4.27	1.09	99.72%
TC-RTK/INS (2)	2.61	0.85	100%	17.77	1.33	98.06%
ARPD-RTK/INS	2.57	0.85	-	4.59	1.06	-

**Table 3:** Trajectory error and fix rate for different RTK algorithms with large pseudorange errors (Unit: m).

Algorithm	Conditional Control Group C			Experimental Group D		
	Max Error	RMSE	Fix Rate	Max Error	RMSE	Fix Rate
Single Epoch RTK	15.811	0.442	87.51%	33.887	2.576	72.85%
TC-RTK/INS (1)	0.026	0.009	100%	3.976	1.208	83.62%
TC-RTK/INS (2)	0.195	0.019	88.78%	1.482	0.073	75.68%
ARPD-RTK/INS	0.021	0.009	-	0.044	0.012	-

**Table 4:** Trajectory error and fix rate for three RTK algorithms on the simulation Scene E (Unit: m).

Algorithm	Max Error	RMSE	Fix Rate
TC-RTK/INS (1)	1.903	0.491	78.07%
TC-RTK/INS (2)	2.050	0.226	69.52%
ARPD-RTK/INS	0.268	0.042	-

**Table 5:** Trajectory error and fix rate for different RTK algorithms on the real dataset (unit: m).

Algorithm	Original Scene F			Frequent Cycle Slips Scene G		
	Max Error	RMSE	Fix Rate	Max Error	RMSE	Fix Rate
TC-RTK/INS (1)	0.351	0.244	90.05%	0.396	0.133	83.58%
TC-RTK/INS (2)	0.818	0.128	85.07%	0.821	0.126	85.07%
ARPD-RTK/INS	0.330	0.079	-	0.376	0.101	-

**Table 6:** Trajectory error and fix rate for different RTK algorithms on the real dataset (unit: m).

Algorithm	Large Noise Scene H			Insufficient Satellite Scene I		
	Max Error	RMSE	Fix Rate	Max Error	RMSE	Fix Rate
TC-RTK/INS (1)	0.665	0.343	86.57%	1.548	0.597	81.59%
TC-RTK/INS (2)	1.253	0.239	72.14%	1.344	0.311	59.20%
ARPD-RTK/INS	0.320	0.082	-	0.458	0.124	-
